# A randomized controlled trial investigating the impact of maternal dietary supplementation with pomegranate juice on brain injury in infants with IUGR

**DOI:** 10.1038/s41598-021-82144-0

**Published:** 2021-02-11

**Authors:** Madeline M. Ross, Sara Cherkerzian, Nicole D. Mikulis, Daria Turner, Julian Robinson, Terrie E. Inder, Lillian G. Matthews

**Affiliations:** 1 Department of Pediatric Newborn Medicine, Brigham and Women’s Hospital, Harvard Medical School, 221 Longwood Ave, Boston, MA 02115 USA; 2 Department of Obstetrics, Brigham and Women’s Hospital, Harvard Medical School, Boston, MA USA; 3 Turner Institute for Brain and Mental Health, Monash University, Melbourne, Australia; 4 Monash Biomedical Imaging, Monash University, Melbourne, Australia

**Keywords:** Randomized controlled trials, Intrauterine growth, Neonatal brain damage

## Abstract

Animal studies have demonstrated the therapeutic potential of polyphenol-rich pomegranate juice. We recently reported altered white matter microstructure and functional connectivity in the infant brain following in utero pomegranate juice exposure in pregnancies with intrauterine growth restriction (IUGR). This double-blind exploratory randomized controlled trial further investigates the impact of maternal pomegranate juice intake on brain structure and injury in a second cohort of IUGR pregnancies diagnosed at 24–34 weeks’ gestation. Ninety-nine mothers and their eligible fetuses (*n* = 103) were recruited from Brigham and Women’s Hospital and randomly assigned to 8 oz pomegranate (*n* = 56) or placebo (*n* = 47) juice to be consumed daily from enrollment to delivery. A subset of participants underwent fetal echocardiogram after 2 weeks on juice with no evidence of ductal constriction. 57 infants (*n* = 26 pomegranate, *n* = 31 placebo) underwent term-equivalent MRI for assessment of brain injury, volumes and white matter diffusion. No significant group differences were found in brain volumes or white matter microstructure; however, infants whose mothers consumed pomegranate juice demonstrated lower risk for brain injury, including any white or cortical grey matter injury compared to placebo. These preliminary findings suggest pomegranate juice may be a safe in utero neuroprotectant in pregnancies with known IUGR warranting continued investigation.

Clinical trial registration: NCT04394910, https://clinicaltrials.gov/ct2/show/NCT04394910, Registered May 20, 2020, initial participant enrollment January 16, 2016.

## Introduction

Intrauterine growth restriction (IUGR), defined as in utero growth that fails to achieve the full endogenous potential of the fetus^1,2^, is a serious complication affecting approximately 10 percent of pregnancies worldwide^3^. Moreover, IUGR is associated with significant risk of perinatal death and neurodevelopmental impairment among surviving infants including cerebral palsy^4–9^. While the etiology of IUGR is complex and multifactorial, it typically refers to growth-restricted fetuses exposed to chronic periods of hypoxia secondary to placental insufficiency or compromised placental blood flow^10,11^. The developing fetal brain is particularly vulnerable to the harmful effects of oxidative stress^10,12^, with the result often similar to that of neurological injury caused by a hypoxic-ischemic event around the time of birth^6,7,10,11,13^. Importantly, for pregnant mothers with a diagnosis of IUGR, few therapeutic options exist. Indeed, management approaches are largely supportive involving monitoring to balance fetal wellbeing and risk of preterm birth^4,14^. Thus, there is an urgent need for preventive measures to protect the developing brain in utero, prior to insult, particularly therapies that are safe and well-tolerated during pregnancy.

Polyphenols are a promising source of potential therapeutic capacity. They are a micronutrient class of antioxidants found naturally in foods like green tea, chocolate, nuts, berries and other fruits including pomegranate^15^. Pomegranate juice is one of the highest polyphenol-containing, commercially available dietary supplements with particularly potent antioxidant capacity due to high bioavailability of biologically active compounds like flavonoids, ellagic acid and ellagitannins. These properties have led to increasing interest in its potential role in the prevention of chronic diseases associated with oxidative stress, such as cardiovascular diseases, cancer, diabetes and neurodegenerative diseases^16–21^, although until recently, no studies have examined the effects of prenatal pomegranate juice consumption in humans^22^.

Pomegranate juice and its derivatives have been studied as potential neuroprotectants in animal models of neurodegeneration, demonstrating benefits in rat models of Alzheimer’s^23^ and more recently, Parkinson’s disease^24^. Previous studies have also demonstrated the therapeutic potential of polyphenol consumption in rat models of stroke^25^ and hypoxic-ischemic injury^26,27^; with rats whose mothers drank pomegranate juice demonstrating markedly reduced brain tissue loss after an ischemic insult^26^. Notably, the degree of neuroprotection increased with increased pomegranate intake^26,27^. Neuroprotectant effects have also been reported in human studies of adult ischemic stroke, with patients randomized to pomegranate pills equivalent to 8 oz daily demonstrating improved cognitive and functional recovery compared with placebo^28^. There have further been reports of memory improvement and increased functional MRI activity during verbal and visual memory tasks in adults following 4 weeks’ daily pomegranate juice consumption^29,30^.

We recently reported differences in white matter microstructure and resting state connectivity within visual networks in IUGR infants born to mothers who consumed pomegranate juice compared to placebo^22^, representing to the best of our knowledge the only study of prenatal pomegranate juice exposure and the developing human brain. In the current study we sought to continue to explore the neuroprotectant potential of pomegranate juice by further investigating associations between pomegranate juice consumption and neonatal brain injury, volumes and white matter microstructure in a second site involving IUGR pregnancies presenting at a major tertiary hospital in Boston, MA. We also addressed gaps from our earlier trial by establishing baseline fetal brain injury prior to juice consumption, and further assessed the safety of high polyphenol intake on fetal ductal constriction^31–33^ using fetal echocardiograms. Neurodevelopmental follow-up at 18–36 months is ongoing and will form the focus of a subsequent publication.

## Results

**Figure 1:**
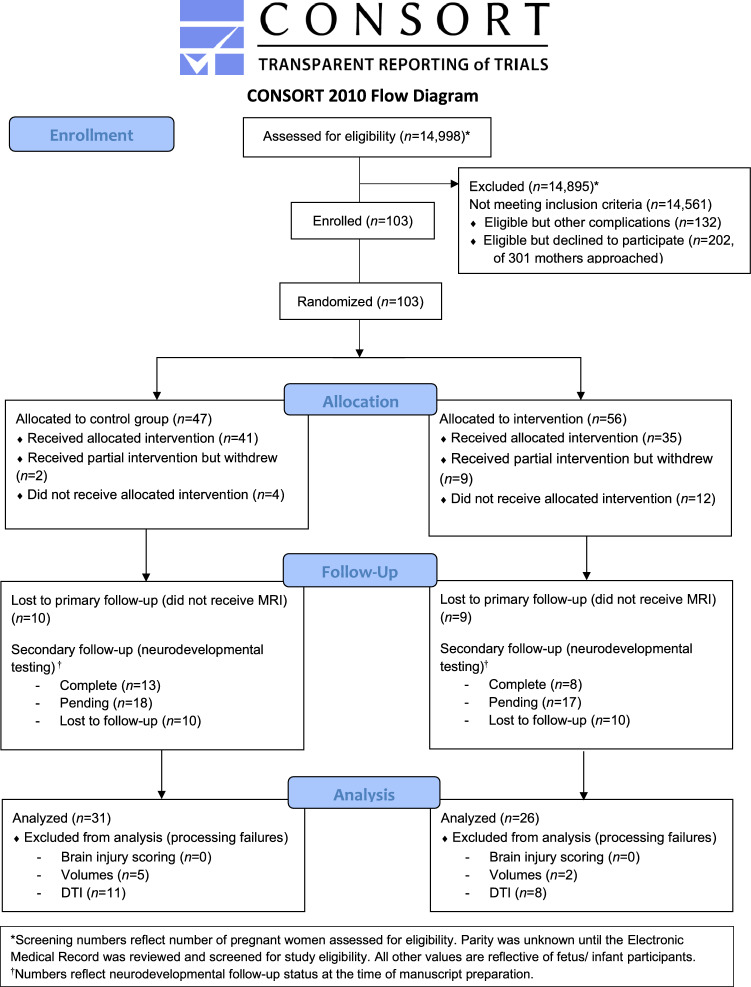
CONSORT participant flowchart.

Ninety-nine mothers and their 103 eligible IUGR fetuses were enrolled and randomly assigned to either treatment (pomegranate juice, *n* = 56) or placebo (*n* = 47) arms (Fig. 1). Of the fetuses randomized to the placebo arm, six (12.8%) were withdrawn from the study: two subjects received partial intervention before withdrawing (*n* = 1 for hyperemesis, *n* = 1 for ductal closure in non-eligible (non-IUGR) co-triplet on first echocardiogram), and four did not receive any intervention (delivered early). Of the 41 fetuses who received the allocated placebo intervention*,* 31 (66%) underwent term-equivalent brain MRI. Among the fetuses randomized to the treatment arm, 21 (37.5%) were withdrawn from the study: nine received partial intervention but withdrew (*n* = 6 aversion to juice, *n* = 1 postnatal genetic anomaly diagnosis, *n* = 1 overwhelmed with pregnancy, *n* = 1 delivering early), and 12 did not receive any intervention (*n* = 6 no longer interested, *n* = 2 congenital anomaly diagnosed after enrollment, *n* = 4 delivered prior to intervention). Of the 35 fetuses who received the allocated treatment intervention*,* 26 (74.3%) underwent term-equivalent brain MRI.

### Baseline characteristics and participant outcomes

There were no differences in estimated fetal weight or IUGR growth percentile between study arms at enrollment (Table 1). The groups were largely similar in other baseline and demographic characteristics in both modified intention-to-treat (mITT) and per protocol (PP) analyses; the latter restricted to participants who adhered to the protocol based on metabolite status. Mothers in the placebo group had significantly higher BMI at enrollment than mothers in the treatment group in both mITT (*p* = 0.009) and PP (*p* = 0.040) analyses. Infants in the treatment group were more likely to be born SGA than those in the placebo group in both mITT (*p* = 0.015) and PP (*p* = 0.031) analyses. This difference is a result of the differential rates of follow-up in the two study arms; with infants lost to follow up (*n* = 46) more likely to be from the treatment group (65.2% vs. 45.6%, *p* = 0.047), compared with infants who underwent infant MRI (mITT total, *n* = 57), and more likely to be appropriate for gestational age (AGA) (62.5% vs. 40.4%, *p* = 0.012); there was no difference in SGA status among total enrolled sample (Table 1). Participants lost to follow-up were also born slightly younger (median (IQR) 35 (33–37) vs 36 (33–37) weeks’ GA, *p* = 0.032), and were more likely to be born preterm (< 37 weeks’ GA), 69.6% vs 58.3%, *p* = 0.037) compared with infants who underwent infant MRI, although these differences did not vary by study arm.

**Table 1:** Baseline maternal and fetal characteristics and neonatal outcomes of study participants.

	Total enrolled		Modified intention-to-treat		Per-protocol	
	Placebo (*n* = 47)	POM (*n* = 56)	Placebo (*n* = 31)	POM (*n* = 26)	Placebo, metabolite − ve (*n* = 17)	POM, metabolite + ve (*n* = 17)
**Baseline characteristics**						
Maternal age (yrs), *mean (SD)*	31.5 (6.8)	29.2 (6.5)	30.5 (5.8)	27.4 (5.5)	30.4 (5.9)	28.0 (5.5)
Maternal BMI, *median (IQR)*	29.9 (26.5, 36.5)	27.7 (24.3, 32.5)*	31.2 (26.5, 37.3)	25.9 (24.2, 30.4)*	31.8 (29.6, 40.7)	26.1 (24.2, 30.4)*
Race, Black *n* (%)	16 (34.0)	21 (37.5)	8 (25.8)	10 (38.5)	5 (29.4)	5 (29.4)
Race, Caucasian *n* (%)	12 (25.5)	15 (26.8)	9 (29.0)	6 (23.1)	2 (11.8)	5 (29.4)
Ethnicity, Hispanic/Latino *n* (%)	13 (27.7)	16 (28.6)	9 (29.0)	8 (30.8)	6 (35.3)	5 (29.4)
Nulliparous, *n (%)*	16 (34.0)	26 (46.4)	12 (38.7)	14 (53.9)	8 (47.1)	9 (52.9)
Current smoking, *n* (%)	2 (4.3)	1 (1.8)	1 (3.2)	0 (0)	0 (0)	0 (0)
Past smoking, *n* (%)	14 (29.8)	12 (21.4)	8 (25.8)	6 (23.1)	2 (11.8)	3 (17.7)
Sickle cell trait, *n* (%)	2 (4.3)	1 (1.8)	1 (3.2)	1 (3.8)	1 (5.9)	1 (5.9)
Past/current diabetes and/or gestational diabetes, *n (%)*	2 (4.3)	5 (8.9)	2 (6.5)	1 (3.8)	0 (0)	1 (5.9)
Multiples, *n (%)*	13 (27.7)	17 (30.4)	9 (29.0)	5 (19.2)	4 (23.5)	4 (23.5)
Gestational age at enrollment (wks), *median (IQR)*	31.4 (29.0, 33.0)	29.9 (27.3, 32.0)	31.9 (29.7, 33.1)	30.7 (27.3, 32.9)	31.4 (29.7, 32.0)	30.6 (27.3, 31.9)
Estimated fetal weight at enrollment (*g*), *mean (SD)*	1242.5 (433.2)	1140.5 (419.3)	1265.4 (431.1)	1157.7 (502.2)	1183.9 (375.0)	1136.1 (490.9)
Growth percentile at enrollment, *median (IQR)*	3 (1, 4)	2 (1, 4)	3 (1, 4)	1.5 (0, 5)	3 (1, 4)	1 (0, 5)
Steroids for fetal lung immaturity, *n* (%)	15 (31.9)	13 (23.2)	9 (29.0)	5 (19.2)	4 (23.5)	3 (17.6)
**Delivery outcomes**						
Gestational age at delivery (wks)*, median (IQR)*	35.0 (34.0, 37.0)	36.0 (33.0, 37.0)	35.0 (34.0, 37.0)	37.0 (34.0, 38.0)	35.0 (34.0, 37.0)	37.0 (36.0, 38.0)
Gestational age at MRI scan, (wks)*, median (IQR)*	40.0 (38.4, 41.4)	39.5 (38.1, 40.0)	40.0 (38.4, 41.4)	39.5 (38.1, 40.0)	39.7 (37.7, 41.4)	39.6 (38.6, 39.9)
Preterm birth < 37 weeks, *n (%)*	29 (61.7)	31 (55.4)	18 (58.1)	10 (38.5)	8 (47.1)	6 (35.3)
Preterm birth < 34 weeks, *n (%)*	10 (21.3)	18 (32.1)	7 (22.6)	5 (19.2)	3 (17.6)	2 (11.8)
Mode of delivery (vaginal), *n (%)*	25 (53.2)	30 (53.6)	15 (48.4)	18 (69.2)	10 (58.8)	12 (70.6)
Meconium stained amniotic fluid, *n (%)*	3 (6.4)	1 (1.8)	2 (6.5)	0 (0.0)	0 (0.0)	0 (0.0)
**Neonatal outcomes**						
Female sex, *n* (%)	27 (57.5)	29 (51.8)	18 (58.1)	11 (42.3)	11 (64.7)	7 (41.2)
Birthweight (g), *mean (SD)*	1993.0 (647.7)	1953.5 (610.0)	2032.8 (617.9)	2018.3 (572.5)	2060.9 (571.4)	2064.2 (476.2)
Birthweight Z-score, *median (IQR)*	− 1.1 (− 1.9, − 0.8)	− 1.35 (− 1.9, − 0.9)	− 1.2 (− 1.9, − 0.8)	− 1.6 (− 2.0, − 1.3)	− 1.1 (− 1.6, − 0.8)	− 1.9 (− 2.1, − 1.4)*
Small for gestational age, *n (%)*	20 (42.6)	30 (53.6)	14 (45.2)	20 (76.9)*	8 (47.1)	14 (82.4)*
APGAR score at 1 min, *median (IQR)*	8 (7, 8)	8 (7, 8)	8 (7, 8)	8 (7, 8)	8 (7, 8)	8 (8, 8)
APGAR score at 5 min, *median (IQR)*	9 (8, 9)	9 (8, 9)	9 (8, 9)	9 (8, 9)	9 (9, 9)	9 (9, 9)
Cord arterial pH^1^, *median (IQR)*	7.2 (7.2, 7.2)	7.3 (7.3, 7.3)	7.2, (7.2, 7.2)	7.3, (7.3, 7.3)	7.2	7.3
Cord arterial base excess^1^, *median (IQR)*	5.2 (4.9, 5.8)	4.1 (2.9, 9.6)	5.8 (4.9, 9.7)	2.3 (1.6, 2.9)	9.7	1.6
Respiratory distress syndrome, *n* (%)	8 (17.0)	12 (21.4)	6 (19.4)	3 (11.5)	3 (17.7)	1 (5.9)

*p < 0.05 POM vs. placebo (placebo = reference).

^1^Cord gas data available for 3 infants in placebo mITT, 2 infants in POM mITT, 1 infant in placebo PP, 1 infant in POM PP.

Among total enrolled sample pH data available for 7 infants in placebo, 7 infants in POM; base excess data available for 6 infants in placebo, 7 infants in POM.

### Brain outcomes

**Figure 2:**
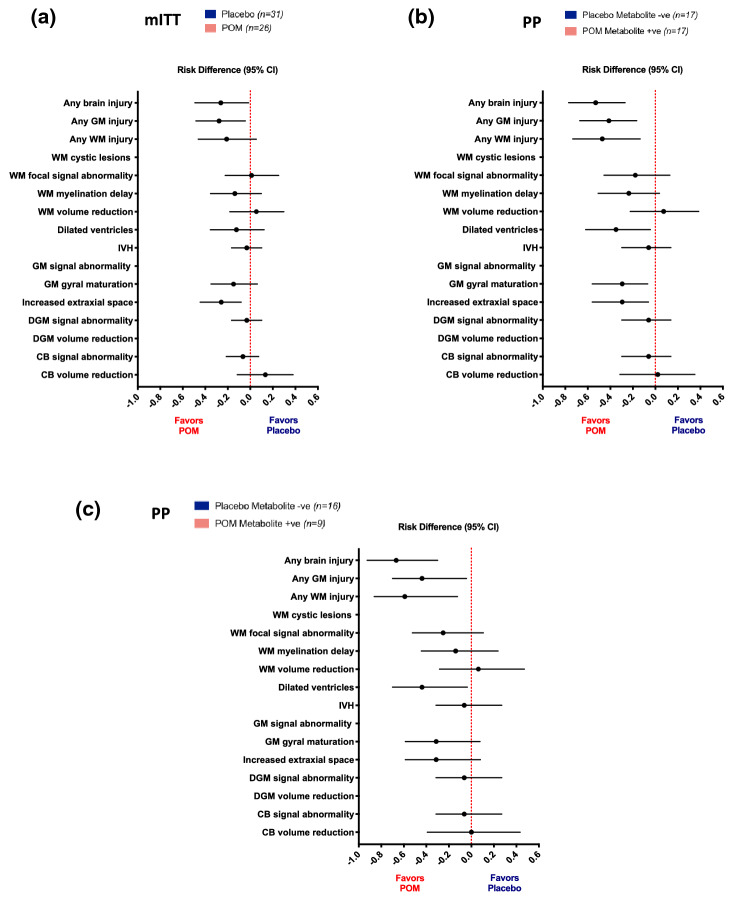
Brain injury risk by study arm on mITT and PP analysis. Risk difference and 95% confidence intervals are shown (Placebo = reference). Lines to the left of 0 favor pomegranate juice, i.e. infants randomized to pomegranate juice demonstrate lower risk of brain injury compared with infants randomized to placebo. Lines that do not cross 0 denote a significant difference in risk, *p* < 0.05. (**a**) Modified intention-to-treat (mITT) analysis. (**b**) PP analysis. (**c**) PP analysis excluding infants born to mothers positive for metabolites at enrollment. *CB* cerebellum; *DGM* deep grey matter; *GM* cortical grey matter; *IVH* intraventricular hemorrhage; *POM* pomegranate; *WM* white matter.

**Figure 3:**
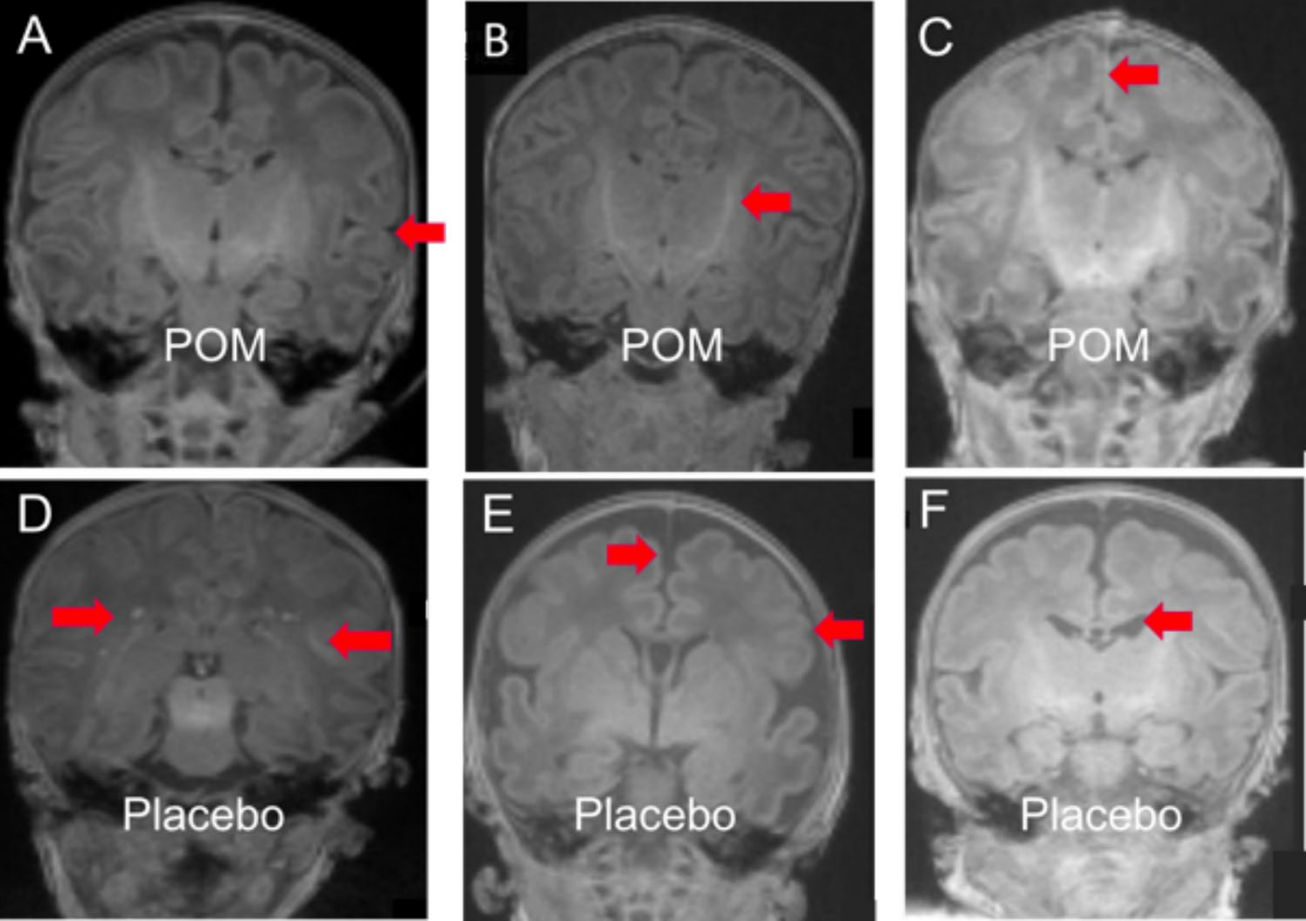
Representative brain MRIs demonstrating Kidokoro injury scoring^34^. *T*_1_-weighted coronal slices from six representative infants exposed in utero to pomegranate juice (*top row*) or placebo (*bottom row*). (**A**–**C**) No injury detected—normal scores for gyral maturation, PLIC myelination, extra-axial space/IHD (arrows). (**D**–**F**) Representative injury categories detected; (**D**)*—*2 points for WM focal signal abnormality (*arrows*); (**E**)—2 points for focal signal abnormality, 1 point for myelination delay, 1 point for gyral maturation delay (*arrow*), 1 point for IHD (*arrow*); (**F**)—1 point for gyral maturation delay, 2 points for IHD, 2 points for lateral ventricle enlargement (*arrow*). *IHD* interhemispheric distance; *PLIC* posterior limb of the internal capsule; *WM* white matter.

There was no evidence of fetal brain injury in any of the participants who underwent fetal MRI prior to starting the allocated juice regimen. We did not observe group differences in total or regional brain volumes, or in DTI measures (Supplementary Tables S1 and S2), however significant risk differences were detected between groups in brain injury measures using the Kidokoro scoring system in both mITT and PP analyses (Table 2, Fig. 2a,b)^34^. *T*_1_-weighted coronal MRIs demonstrating representative brain injury scoring are shown in Fig. 3.

**Table 2:** Neonatal brain injury frequency and risk comparison by study arm.

Modified intention-to-treat	Placebo (*n* = 31)		POM (*n* = 26)		Group comparison	
	Grade 0	Grade ≥ 1	Grade 0	Grade ≥ 1	Risk difference^1^ (95% CI)	Relative risk^2^ (95% CI)
WM cystic lesions	31 (100)	0 (0)	26 (100)	0 (0)	N/A	N/A
WM focal signal abnormality	23 (74.2)	8 (25.8)	19 (73.1)	7 (26.9)	0.01 (− 0.22, 0.25)	1.04 (0.38, 2.63)
WM myelination delay	22 (71.0)	9 (29.0)	22 (84.6)	4 (15.4)	− 0.14 (− 0.35, 0.10)	0.53 (0.08, 1.48)
WM volume reduction^3^	24 (77.4)	7 (22.6)	18 (72.0)	7 (28.0)	0.05 (− 0.18, 0.30)	1.24 (0.44, 3.49)
Dilated ventricles^3^	21 (67.7)	10 (32.3)	20 (80.0)	5 (20.0)	− 0.12 (− 0.36, 0.12)	0.62 (0.17, 1.57)
GM signal abnormality	31 (100)	0 (0)	26 (100)	0 (0)	N/A	N/A
GM gyral maturation	24 (77.4)	7 (22.6)	24 (92.3)	2 (7.7)	− 0.15 (− 0.35, 0.06)	0.34 (0.04, 1.39)
Increased extra-axial space^3^	23 (74.2)	8 (25.8)	25 (100)	0 (0)	− 0.26 (− 0.45, − 0.08)*	0.75 (0.60, 0.93)*
DGM signal abnormality	30 (96.8)	1 (3.2)	26 (100)	0 (0)	− 0.03 (− 0.17, 0.10)	0.97 (0.89, 1.07)
DGM volume reduction^3^	31 (100)	0 (0)	25 (100)	0 (0)	N/A	N/A
Cerebellar signal abnormality	29 (93.5)	2 (6.5)	26 (100)	0 (0)	− 0.07 (− 0.21, 0.07)	0.94 (0.84, 1.05)
Cerebellar volume reduction^3^	24 (77.4)	7 (22.6)	16 (64.0)	9 (36.0)	0.13 (− 0.12, 0.38)	1.59 (0.64, 4.07)
IVH	30 (96.8)	1 (3.2)	26 (100)	0 (0)	− 0.03 (− 0.17, 0.10)	0.97 (0.89, 1.07)
Any WM injury	9 (29.0)	22 (71.0)	13 (50.0)	13 (50.0)	− 0.21 (− 0.46, 0.05)	0.71 (0.38, 1.10)
Any GM injury	20 (64.5)	11 (35.5)	24 (92.3)	2 (7.7)	− 0.28 (− 0.48, − 0.05)*	0.22 (0.02, 0.89)*
Any brain injury	5 (16.1)	26 (83.9)	11 (42.3)	15 (57.7)	− 0.26 (− 0.49, − 0.02)*	0.69 (0.42, 0.98)*

Per protocol	Placebo, metabolite − ve (*n* = 17)		POM, metabolite + ve (*n* = 17)		Group comparison	
	Grade 0	Grade ≥ 1	Grade 0	Grade ≥ 1	Risk difference^1^ (95% CI)	Relative risk^2^ (95% CI)
WM cystic lesions	17 (100)	0 (0)	17 (100)	0 (0)	N/A	N/A
WM focal signal abnormality	12 (70.6)	5 (29.4)	15 (88.2)	2 (11.8)	− 0.18 (− 0.46, 0.13)	0.40 (0.06, 1.83)
WM myelination delay	12 (70.6)	5 (29.4)	16 (94.1)	1 (5.9)	− 0.24 (− 0.51, 0.04)	0.20 (0.03, 1.54)
WM volume reduction^3^	14 (82.4)	3 (17.7)	12 (75.0)	4 (25)	0.07 (− 0.22, 0.39)	1.42 (0.34, 8.05)
Dilated ventricles^3^	10 (58.8)	7 (41.2)	15 (93.8)	1 (6.3)	− 0.35 (− 0.62, − 0.04)*	0.15 (0.01, 0.87)*
GM signal abnormality	17 (100)	0 (0)	17 (100)	0 (0)	N/A	N/A
GM gyral maturation	12 (70.6)	5 (29.4)	17 (100)	0 (0)	− 0.29 (− 0.56, − 0.07)*	0.71 (0.52, 0.98)*
Increased extra-axial space^3^	12 (70.6)	5 (29.4)	16 (100)	0 (0)	− 0.29 (− 0.56, − 0.06)*	0.72 (0.52, 0.98)*
DGM signal abnormality	16 (94.1)	1 (5.9)	17 (100)	0 (0)	− 0.06 (− 0.30, 0.14)	0.94 (0.80, 1.11)
DGM volume reduction^3^	17 (100)	0 (0)	16 (100)	0 (0)	N/A	N/A
Cerebellar signal abnormality	16 (94.1)	1 (5.9)	17 (100)	0 (0)	− 0.06 (− 0.30, 0.14)	0.94 (0.80, 1.11)
Cerebellar volume reduction^3^	11 (64.7)	6 (35.3)	10 (62.5)	6 (37.5)	0.02 (− 0.31, 0.35)	1.06 (0.35, 3.22)
IVH	16 (94.1)	1 (5.9)	17 (100)	0 (0)	− 0.06 (− 0.297, 0.138)	0.94 (0.80, 1.11)
Any WM injury	3 (17.6)	14 (82.4)	11 (64.7)	6 (35.3)	− 0.47 (− 0.73, − 0.14)*	0.43 (0.17, 0.83)*
Any GM injury	10 (58.8)	7 (41.2)	17 (100)	0 (0)	− 0.41 (− 0.67, − 0.17)*	0.60 (0.40, 0.89)*
Any brain injury	0 (0)	17 (100)	9 (52.9)	8 (47.1)	− 0.53 (− 0.77, − 0.27)*	0.47 (0.23, 0.72)*

Group summaries are *n (%).*

Group comparisons tested using exact methods due to small sample size (i.e. cell counts < 5).

*p < 0.05.

^1^Absolute effect size reported as the risk difference (placebo = reference). Corresponding 95% confidence intervals are reported.

^2^Relative effect size reported as relative risk (placebo = reference). For analyses with zero in one or more cells, 0.5 was added to each cell prior to calculation of the relative risk. Corresponding 95% confidence intervals are reported.

^3^Metric-based data unavailable for 1 POM infant (not possible due to incomplete acquisition).

*DGM* deep grey matter; *GM* cortical grey matter; *IVH* intraventricular hemorrhage; *POM* pomegranate; *WM* white matter.

Infants randomized to pomegranate juice were less likely to demonstrate any brain injury compared to those randomized to placebo (mITT 57.7% vs. 83.9%; PP 47.1% vs. 100%), with approximately 30 to 50% lower risk of any brain injury in mITT and PP analyses, respectively (Table 2, Fig. 2a,b, Supplementary Tables S4-S6). More specifically, infants born to mothers receiving pomegranate juice demonstrated lower risk of any cortical grey matter injury, including lower risk of increased extra-axial space (interhemispheric distance, IHD) and gyral maturation delay, relative to infants in the placebo group. Infants exposed to pomegranate juice further demonstrated lower risk of any white matter injury, including lower risk of enlarged lateral ventricles compared with placebo infants. Although there were no statistically significant differences between groups for several white matter injury components, including cysts and focal signal abnormalities, infants in the treatment arm demonstrated notably lower frequency of delayed myelination particularly in PP analyses (5.9% vs. 29.4%) compared with placebo (Table 2, Fig. 2b).

While we identified differences in SGA and maternal BMI by study arm, brain injury analyses were not adjusted for either as there was no association between these covariates and injury outcome, with the exception of an association between maternal obesity (BMI > 30) and extra-axial space and dilated ventricles in mITT and PP analyses, respectively. To assess maternal obesity as a potential confounder in the association between study arm and extra-axial space and dilated ventricles in mITT and PP analyses, we performed supplemental stratified analysis by obesity status and observed greater frequency of injury among infants whose mothers were randomized to placebo compared to those randomized to pomegranate juice, irrespective of maternal obesity status (Supplementary Table S7), consistent with results from unadjusted analyses.

As part of the injury scoring system^34^, continuous measures were first generated for white matter (biparietal diameter), deep grey matter (total basal ganglia area) and cerebellar (trans-cerebellar diameter) volume reduction, and for ventricular dilatation (ventricular diameter) and extra-axial cerebrospinal fluid-filled space (IHD) prior to conversion to their corresponding scores. We performed supplemental analysis exploring group differences in these continuous measures using median regression and found no differences by study arm (Supplementary Table S3). Further exploration of the variable distributions by study arm indicated that, for increased extra-axial space and enlarged ventricles, the placebo arm presented longer right tails in its distributions relative to the treatment arm, with greater differences between study arms observed at upper quartiles. Furthermore, while we observed differences in the corresponding categorical measures, injury score cut-offs for ventricular (one side > 7.5 mm) and IHD (> 4 mm) measures^34^ in the placebo arm were located within the right tail of these distributions, such that we did not observe differences in the central tendencies of these distributions (Supplementary Table S3).

### Compliance

There were no significant differences in the time period on juice or in the recorded days of juice consumption between study arms (Table 3). Maternal metabolite presence (either UA or DMEAG present in blood or urine) at enrollment also did not differ between groups in mITT analysis, with 27.6% and 32.0% of mothers being metabolite positive before starting the juice regimen in the placebo and treatment arms, respectively. Of note, in PP analysis, there was a higher prevalence of maternal metabolite presence at enrollment in the treatment arm than in placebo (50.0% vs. 5.9%, *p* = 0.007, Table 3).

**Table 3:** Measures of compliance.

	Modified intention-to-treat							Per protocol						
	Placebo (*n* = 31)	POM (*n* = 26)	Absolute effect size^1^	Relative effect size^2^	SE^2^	$z$/${\chi }^{2}$	*P* value	Placebo, metabolite − ve (*n* = 17)	POM, metabolite + ve (*n* = 17)	Absolute effect size^1^	Relative effect size^2^	SE^2^	$z$/ ${\chi }^{2}$	*P* value
Time period on juice in days, *median (IQR)*	19 (11, 34)	28.5 (19, 46)	9.50	0.37	2.43	1.73	0.083	28 (13, 39)	33 (25, 47)	5.00	0.34	3.23	1.19	0.234
Recorded days of consumption, *median (IQR)*^3^	17 (10.5, 30.5)	35 (22.5, 47.5)	18.00	0.52	3.38	1.85	0.064	27 (15.5, 34.5)	36 (25, 52.5)	9.00	0.57	4.13	1.03	0.301
Maternal UA or DMEAG plasma at enrollment, *n (%)*	2 (6.9)^a^	3 (13.0)^d^	6.15%	1.89	0.08		0.644	1 (5.9)	3 (21.4)^f^	15.55%	3.64	0.12		0.304
Maternal UA or DMEAG urine at enrollment, *n (%)*	8 (27.6)^a^	8 (32.0)^c^	4.41%	1.16	0.12	0.125	0.723	1 (5.9)	8 (50.0)^g^	44.12%	8.50	0.16		0.007
Positive for metabolites in plasma or urine at enrollment, *n (%)*	8 (26.7)^b^	8 (32.0)^c^	5.33%	1.20	0.12	0.188	0.664	1 (5.9)	8 (50.0)^g^	44.12%	8.50	0.16		0.007
Maternal UA or DMEAG plasma at delivery, *n (%)*	3 (12.0)^c^	9 (40.9)^e^	28.91%	3.41	0.13		0.042	0 (0)^h^	9 (60.0)^h^	60.00%	19.00	0.16		< .001
Maternal UA or DMEAG urine at delivery, *n (%)*	13 (41.9)	16 (64.0)^c^	22.06%	1.53	0.13	2.699	0.100	0 (0)	16 (94.1)	94.12%	33.00	0.17		< .001
Cord UA or DMEAG at delivery, *n (%)*	2 (8.7)^d^	9 (39.1)^d^	30.43%	4.50	0.13		0.035	0 (0)^i^	9 (64.3)^i^	64.29%	19.00	0.17		< .001
Positive for metabolites in cord blood or maternal blood or urine at delivery, *n (%)*	14 (45.2)	17 (65.4)	20.22%	1.45	0.13	2.331	0.127	0 (0)	17 (100.0)	100.00%	35.00	0.17		< .001

Group comparisons evaluated using Wilcoxon rank sum and *χ*^2^ tests, as appropriate.

Fisher’s exact test (2-sided) used to compare proportions by group where expected cell counts < 5.

^1^Absolute effect size calculated as the median difference for continuous variables, and the risk difference (%) for categorical variables (placebo = reference).

^2^Relative effect size calculated as Cohen’s *d* for continuous variables, and the relative risk for categorical variables (placebo = reference). For analyses with zero in one or more cells, 0.5 was added to each cell prior to calculation of the relative risk and its standard error (SE). Corresponding SE are reported.

^3^Logbook data available for 19 infants in placebo mITT, 11 infants in POM mITT, 11 infants in placebo PP, 8 infants in POM PP.

^a^*n* = 29; ^b^*n* = 30; ^c^*n* = 25; ^d^*n* = 23; ^e^*n* = 22; ^f^*n* = 14; ^g^*n* = 16; ^h^*n* = 15; ^i^*n* = 14.

*DMEAG* dimethylellagic acid glucuronid; *IQR* interquartile range; *POM* pomegranate; *SE* standard error; *UA* urolithin A.

In mITT analysis, there was higher metabolite detection at delivery in the treatment arm compared to placebo in plasma (40.9% vs. 12%, *p* = 0.042), urine (64% vs. 41.9%, *p* = 0.1), and cord blood (39.1% vs. 8.7%, *p* = 0.035). However, a large number of placebo participants (45.2%) were positive for metabolites at delivery, detected in either cord blood, maternal blood or maternal urine.

Due to the variability of metabolite presence both at enrollment and delivery between groups, PP analyses were performed using strict metabolite criteria, which included only placebo participants who were metabolite-negative at delivery, and treatment participants who were metabolite-positive at delivery.

To further address the difference in maternal metabolite presence between study arms at enrollment in PP analyses and any potential confounding due to non-random baseline metabolite positivity, we reran PP analyses for brain injury outcomes, excluding the 9 infants born to mothers who were positive for metabolites at enrollment. While associations for gyral maturation, extra-axial space and lateral ventricles lost statistical significance, this was due to a reduction in power, with no change in the number of infants with injury in either study arm (5 (31.3%) placebo infants with gyral maturation delay, and 5 (31.3%) placebo infants with increased extra-axial space compared with 0 infants with either injury in the treatment arm). Findings for white matter injury, cortical grey matter injury and any brain injury further remained unchanged, maintaining statistical significance (Fig. 2c).

### Safety assessment

Safety was assessed in infants born to mothers who completed either full or partial juice regimen (total *n* = 87). No differences were observed between groups with respect to any complications (Table 4). As part of Phase 1, 17 participants (8 in placebo, 9 in treatment) underwent two fetal echocardiograms: one before starting the juice regimen, and one approximately two weeks after starting daily juice consumption in order to investigate whether a diet rich in polyphenol-rich foods can lead to fetal ductal constriction^31–33^. There was no evidence of ductal constriction in either the treatment or placebo groups in association with juice consumption. As no adverse events were reported, no stopping rules were implemented.

**Table 4:** Safety measures.

Complication	Placebo (*n* = 43)	POM (*n* = 44)	Risk difference^1^ (%)	Relative risk^2^	SE^2^	${\chi }^{2}$	*P* value
NICU admission, *n* (%)	22 (51.2)	25 (56.8)	5.66	1.11	0.11	0.28	0.597
Respiratory distress, *n* (%)	7 (16.3)	6 (13.6)	− 2.64	0.84	0.08	0.12	0.730
Resuscitation at delivery, *n* (%)	2 (4.7)	2 (4.6)	− 0.11	0.98	0.04		1.000
Intraventricular hemorrhage, *n* (%)	1 (2.3)	0 (0)	− 2.33	0.33	0.03		0.494
Sepsis, *n* (%)	0 (0)	1 (2.3)	2.27	2.93	0.03		1.000
Necrotizing enterocolitis, *n* (%)	0 (0)	0 (0)	0.00	0.98	0.02		N/A

Group comparisons evaluated using Wilcoxon rank sum and *χ*^2^ tests, as appropriate.

Fisher’s exact test (2-sided) used to compare proportions by group where cell counts < 5.

^1^Absolute effect size calculated as the risk difference (%, placebo = reference).

^2^Relative effect size calculated as the relative risk for categorical variables (placebo = reference). For analyses with zero in one or more cells, 0.5 was added to each cell prior to calculation of the relative risk and its standard error (SE). Corresponding SE are reported.

*NICU* neonatal intensive care unit; *POM* pomegranate; *SE* standard error.

## Discussion

In this double-blind, exploratory randomized controlled trial we present findings suggesting in utero exposure to polyphenol-rich pomegranate juice may reduce the risk of perinatal brain injury in the at-risk infant with IUGR. No injury was observed on fetal MRI in the subset of infants who underwent fetal MRI prior to the start of juice consumption, indicating that the reported brain injury occurred during the third trimester, over the course of the juice regimen. Our findings therefore suggest that maternal pomegranate juice intake may have a beneficial effect during a period of marked brain vulnerability such as late gestation. Furthermore, while recent reports have suggested that a polyphenol-rich diet can lead to fetal ductal constriction^31–33^, we did not observe any instances of ductal constriction related to maternal intake of 8 oz pomegranate juice, suggesting its daily consumption is safe and without attributable side effects.

Although the underlying mechanisms are not yet fully understood, pomegranate juice and its derivatives have demonstrated anti-inflammatory and anti-apoptotic properties^35^, and have been shown to act as cytoprotective agents through direct scavenging of reactive oxygen species, and increased antioxidant enzymatic response^36^, suggesting multiple potential modes of action. While the relationships between impaired placental perfusion, oxygen and nutrient transfer disturbances and subsequent brain abnormality in IUGR remain unclear, it is possible that the observed brain injury may reflect a primary or secondary dysmaturational event. We had initially postulated that pomegranate juice may exert its protective effect on the placenta, leading to improved outcomes for the fetus, including reduced brain injury. Indeed, using a hypoxia-induced growth restriction mouse model, Chen et al*.* reported decreased placental heat shock protein expression and apoptosis in pregnant dams who received pomegranate juice compared to glucose^37^. Notably, pregnant dams consumed juice prior to and in conjunction with hypoxic exposure, suggesting that antecedent pomegranate juice may be critical for placental protection. In our study, however, IUGR was not diagnosed until late in the second trimester, such that by the time the juice regimen is started, it is likely too late post-implantation to change the placental villous structure. Indeed, secondary analysis of our earlier cohort has shown no difference in placental pathology between pomegranate and placebo arms^38^, suggesting the lower injury risks reported in the current study may reflect a distinct cerebral effect of pomegranate juice. Future assessment of placental morphometry and pathology will provide added insight into potential differential placental and cerebral protection.

The pathophysiology of perinatal brain injury, including that following IUGR, is complex with varying degrees of grey and white matter involvement, depending on the nature, location, timing and severity of the injury^39–43^. Our findings for lower risk of white matter injury associated with pomegranate juice exposure may suggest a beneficial effect on pre-oligodendrocyte (pre-OL) maturation, a dominant feature of cerebral white matter development during late gestation^44,45^, and the period during which enrolled mothers consumed juice. Pre-OL vulnerability to oxidative attack is well established, and has been postulated to relate to the lack of white matter antioxidant capacity before birth, rendering the immature white matter susceptible to free radical toxicity as the fetus transitions to an oxygen-rich postnatal environment^13,46^. Thus, our white matter findings appear to be consistent with potential benefit via reduced susceptibility to redox status perturbations and improved oligodendrocyte maturation or sparing. Relatedly, while we did not observe significant differences in myelination, likely due to sample size limitations, infants in the treatment arm demonstrated notably lower frequency of delayed myelination on PP analyses.

Our findings of lower grey matter injury risk may reflect associated sparing of secondary axonal disturbance and impaired gray matter maturation^45,47^. Cerebral cortex undergoes dramatic expansion during the last trimester^48–51^ characterized by increased complexity of neuronal processes, synaptogenesis, and tertiary folding^49,52^. Cortical developmental vulnerability including impaired gyrification has been demonstrated in growth-restricted fetuses and preterm infants with IUGR^6,7,53,54^, and has been linked to subplate neurons beneath the developing cortical plate^41,55–58^ and their susceptibility to hypoxic injury and excitotoxic cell death^59^. Furthermore, and similar to pre-OLs, peak subplate neuron development occurs between 24–32 weeks’ gestation before undergoing programmed cell death^60,61^. Of note, our previous findings of greater functional connectivity in visual networks would appear to lend support to a beneficial effect at the level of subplate neurons^22^, given their implication in normal visual cortical development^62^ and roles in early establishment of thalamocortical connections to visual cortex^56,57,62^. Our findings for gyral maturation also appear to be consistent with potential sparing of upper cortical neurons over this period; given disturbances in late migration of GABAergic neurons destined for upper cortical layers have been proposed to lead to gyral maturation delay^47,63^.

The observed lower frequency of enlarged ventricles and increased extra-axial space may reflect associated cortical tissue sparing. Of note, brain injury scoring in the current study incorporated both qualitative signal abnormality scores as well as scores based on continuous quantitative biometrics^34^. For ventricular and IHD measures, differences between study arms observed using categorical scores appeared to be masked in analyses using continuous measures, because the corresponding injury scoring cut-offs were located within the right tail of these distributions. This may explain the lack of observable group differences in our earlier trial where brain metric measures were assessed separately from signal abnormality measures^22^.

Given the exploratory nature of the current study, larger controlled trials are needed to better assess the aforementioned relationships and speculative mechanisms before definitive conclusions can be drawn. Longitudinal studies to track the pre-post-conception effects of pomegranate juice on injury evolution, brain development and linear growth in the growth-restricted population may also be merited. The latter is of particular interest given a recent animal study suggesting maternal consumption of pomegranate juice may not be beneficial for fetal growth restriction^64^. While the authors reported reduced litter size and biometric measurements, these findings are difficult to extrapolate to the more complex etiology of human IUGR. Importantly, they did not observe differences in fetal weight related to pomegranate juice consumption^64^. In line with this, our observed group differences in SGA by study arm were due to a differential loss to follow up of AGA infants in the treatment arm, with no group differences in SGA among total enrolled sample, suggesting the higher frequency of SGA in the treatment arm was not due to a pomegranate juice effect.

While the results of this study appear to be consistent with the hypothesized therapeutic potential of prenatal pomegranate juice, there are several limitations that warrant consideration. We acknowledge limitations with our trial being retrospectively registered and study protocol changes occurring subsequent to randomization, including cessation of fetal MRI acquisition due to scheduling difficulties and stricter inclusion cut-off for IUGR early; however, protocol variations occurred while maintaining blinding to accumulating data. In addition to further challenges related to the small sample size and participant attrition, there are difficulties related to measures of metabolite bioavailability^65^; estimates based on plasma pharmacokinetics may not yield accurate quantitative measures of absorption; however, we attempted to address this by also capturing urinary excretion^66^, a strength over our earlier trial. We further attempted to address compliance issues through strict PP analyses focusing on metabolite positivity rather than group allocation alone with our results largely consistent across all analyses. We observed group differences in baseline metabolite status suggesting non-random distribution of starting polyphenol levels from non-pomegranate dietary sources^65,67,68^ such as green tea, chocolate, nuts, berries^15^; however, we accounted for this in further analysis excluding infants born to mothers positive for metabolites at enrollment; with results again remaining largely unchanged. Nonetheless, we acknowledge that potential bias arising from participant exclusion cannot be excluded in our PP analyses. Another limitation relates to the lack of resting state functional MRI due to clinical scanner time and data quality constraints, so we were unable to further investigate our previously reported functional connectivity findings^22^. Finally, future trials may benefit from incorporating additional criteria for IUGR diagnosis beyond estimated fetal weight; although in the current study, we used a more conservative cutoff for inclusion (< 5th percentile) than that widely used to ensure infants were truly growth restricted, as evidenced by average birthweights of approximately 2 kg.

## Conclusion

In this double-blind, exploratory randomized controlled trial, we present further preliminary findings suggestive of potential in utero neuroprotectant effects of maternal dietary supplementation with pomegranate juice. We report decreased brain injury risk in IUGR infants exposed to pomegranate juice compared with placebo. Importantly, we demonstrate that 8 oz daily of polyphenol-rich pomegranate juice during pregnancy does not increase risk for fetal ductal constriction. Together with our earlier work, these findings warrant continued investigation and suggest secondary studies using advanced quantitative techniques such as cortical surface analysis and non-tensor based diffusion analysis may be needed to better untangle brain structural alterations associated with the observed brain injury differences. Such studies could aid in identifying brain outcome measures that may be more sensitive to the potential neuroprotectant effects of pomegranate juice. Neurodevelopmental follow-up of this cohort is ongoing and will provide further insight into the potential functional correlates and long-term clinical implications of prenatal dietary supplementation with pomegranate juice.

## Methods

### Trial design and participants

This was a double-blind, exploratory randomized controlled trial of maternal POM consumption during pregnancy, and represents a follow-up to a previously published study^22^, involving a second population of IUGR pregnancies presenting at a major tertiary hospital in Boston, MA. Study staff screened high risk ultrasound reports of expectant mothers receiving prenatal care at Brigham and Women’s Hospital between October 2015 and March 2020. Due to a period of rapid study staff turnover in the initial stages of study set-up, the trial was inadvertently not prospectively registered, but has since been registered to clinicaltrials.gov (NCT04394910). Initial inclusion criteria were: 1) fetal diagnosis of IUGR < 10th percentile on the Hadlock growth curve^69^ and at least one of the following: 2) concern over umbilical artery doppler flow for gestational age as per standard clinical Brigham and Women’s Hospital guidelines, 3) reduction in amniotic fluid volume. Following enrollment of the fourth participant, the inclusion criteria were amended to ensure that eligible participants were truly growth restricted, and not simply constitutionally small fetuses^11,70,71^. The doppler flow and amniotic fluid criteria were also eliminated because these conditions were not routinely clinically reported, resulting in recruitment difficulties. The amended inclusion criteria were: 1) fetal diagnosis of IUGR defined by estimated fetal weight < 5th percentile for gestational age on the Doubilet growth curve^72^; 2) 24–34 weeks’ gestation based on ultrasound or reliable clinical dating by ACOG standards^73^. Exclusion criteria were: 1) multiple congenital anomalies; 2) known fetal chromosomal disorder; 3) maternal illicit drug or alcohol intake. Notably, the first four participants enrolled under the initial inclusion criteria fulfilled the updated recruitment criteria. All protocols were in accordance with the 1964 Helsinki declaration and its amendments or comparable ethical standards. The study was approved by the Partners Healthcare Institutional Review Board. Written informed consent was obtained for all participants.

### Enrollment study visits

Maternal blood and urine samples were collected at enrollment to measure baseline metabolite levels. Samples were sent to the University of California, Los Angeles Center for Human Nutrition for liquid chromatography-tandem mass spectrometry (LCMS/MS) analysis of pomegranate juice metabolites, Urolithin A (UA) and dimethylellagic acid glucuronide (DMEAG). Sociodemographic, health status, and pregnancy information of consented participants were collected by review of the electronic medical record.

The first 31 mothers (34 infants) were recruited as part of a Phase 1 study to assess the effect of high polyphenol intake on fetal ductal constriction, and were scheduled to receive two fetal echocardiograms, the first at enrollment before commencing juice consumption and the second after two weeks of the daily juice regimen.

A subset of participants also received fetal MRI (*n* = 44) prior to commencing juice consumption to establish baseline brain injury; fetal MRIs were subsequently eliminated due to difficulty in scheduling, and following preliminary analyses revealed no fetal brain injury in any of the participants who underwent fetal MRI prior to starting the allocated juice regimen.

### Randomization

Participants were block randomized by random number generator and blinded envelope in a 1:1 ratio to a daily regimen of either 8 oz of 100% pomegranate juice (POM Wonderful, Los Angeles, CA) or a polyphenol-free control beverage matched for color, taste and calorie content. Total polyphenols were determined by the Folin-Ciocalteu method calibrated by a gallic acid standard curve and reported as gallic acid equivalents (GAE)^15,74,75^. Pomegranate juice (16 ± 0.2° Brix) contained no less than 700 mg GAE per 8 oz serving, and placebo (16 ± 0.2° Brix) contained no more than 38 mg GAE per serving. Juice was distributed as 8 oz bottles labeled A or B; bottles were visually indistinguishable such that the investigative team, participants, and care providers remained blinded to group allocation. Participants were instructed to begin juice consumption on the day of initial study visit, following collection of enrollment blood and urine samples, and first echocardiogram and fetal MRI when applicable, through to delivery.

### Follow-up and compliance

Participants were followed up from enrollment until delivery. Juice consumption was tracked in two ways: participants kept a daily diary documenting the number of days of juice consumption, and study staff recorded juice consumption on a weekly basis while delivering juice at regular clinical prenatal appointments. Maternal blood and urine, and cord blood were collected at delivery and analyzed to determine change in polyphenol levels from baseline and to confirm transfer of pomegranate metabolites. Information regarding mode and type of delivery, labor and delivery complications, and neonatal outcomes were recorded from the electronic medical record. Clinically stable infants underwent term equivalent brain MRI. Formal neurodevelopmental follow-up at 18–36 months is currently ongoing.

### Image acquisition

Infants were scanned without sedation at 37–41 weeks’ postmenstrual age on a 3 T Siemens Trio scanner (Erlangen, Germany) at Brigham and Women’s Hospital. Images included a turbo spin echo *T*_2_-weighted sequence (TR/TE 8630/133 ms, FOV = 190 × 190, matrix = 192 × 192, refocusing flip angle = 160°, voxel size 1 × 1 × 1 mm^3^) and diffusion data obtained using a 2D spin-echo echo-planar-image (EPI) sequence with 30 gradient directions, *b* value = 1000 s/mm^2^, 1 *b* = 0, and spatial resolution 2 × 2 × 2 mm^3^. Images were interpreted by pediatric neuroradiologists (Drs. Edward Yang and Ellen Grant) and a neonatologist experienced in neuroradiology and brain injury scoring (TEI).

### MR image analysis

All term-equivalent MRI analysis was performed blinded to treatment group. Brain injury was scored on *T*_1_- and *T*_2_- weighted images using the Kidokoro scoring system^34^, capturing signal abnormality and volume reduction measures in white matter, grey matter, deep grey matter and cerebellum, as well as measures of gyral maturation delay, ventricular dilatation, increased CSF-filled extra-axial space, and intraventricular hemorrhage. Total and regional brain volumes were generated using MANTiS^76^. Diffusion data was processed using a tensor model. Images were distortion and motion corrected using FSL^77^. Regions of interest were manually drawn on each brain^78^ using FSLview (Mayo Clinic, Rochester, MN) in the bilateral anterior and posterior limbs of the interior capsule (ALIC, PLIC), optic radiations, frontal and occipital lobes, to generate diffusion tensor imaging (DTI) measures of fractional anisotropy (FA), mean (MD), radial (RD) and axial diffusivity (AD). Twenty subjects were excluded from DTI analysis due to processing failure (12 placebo, 8 treatment). Six subjects were excluded from MANTiS analysis due to processing failure (4 placebo, 2 treatment).

### Safety assessment

Safety was assessed via review of the electronic medical record for frequency of adverse neonatal outcomes, including neonatal intensive care unit (NICU) admission, respiratory distress, resuscitation at delivery, intraventricular hemorrhage on clinical ultrasound^79^, sepsis, and necrotizing enterocolitis. Assessors were unaware of study-group allocation. Of the 31 mothers (34 infants) recruited as part of Phase 1 to assess the effect of high polyphenol intake on fetal ductal constriction, 17 completed two fetal echocardiograms according to protocol specifications, revealing no evidence of fetal duct constriction. Of the remaining 17 fetuses who did not receive two fetal echocardiograms, 8 were removed from the study (2 delivered before juice commencement, 1 withdrew due to ductal closure in non-eligible (non-IUGR) co-triplet on first echo before juice commencement, 3 (triplets) withdrew due to maternal migraines triggered by juice, 2 withdrew due to maternal change of mind), 5 completed the first fetal echo but delivered within two weeks of juice consumption, i.e. before the second echo could be performed, and 5 did not undergo either echocardiogram due to scheduling complications.

### Outcomes measures

#### Primary outcomes

Term-equivalent MRI measures of brain structure and injury included total and regional brain volumes, brain injury scoring, and region-of-interest diffusion measures of FA, MD, AD, and RD.

#### Secondary outcomes

Measures of treatment compliance included time period on juice, number of recorded days of juice consumption, maternal blood and urine metabolite concentrations (enrollment and delivery) and cord blood metabolite concentrations. Safety outcomes included NICU admission, respiratory distress, resuscitation, IVH, sepsis and NEC.

Neurodevelopmental testing is currently ongoing using the Bayley III exam to assess cognitive, gross and fine motor, and language outcomes at 18–36 months; this will form the focus of a subsequent publication.

Measures of placental morphometry and pathology were also collected and will be reported in future secondary analysis of this cohort.

### Statistical analysis

Power calculations were based on effect sizes with regard to the primary brain outcome measures of a comparable study in high-risk infant populations, which reported medium to large standardized effect sizes between 0.7–1.3^80^. Accounting for the feasibility of our study, our enrollment of 103 pregnant mothers (*n* = 47 placebo; *n* = 56 POM) yields > 90% power based on a two-tailed test of significance (α = 0.05) to detect an effect size of 0.7, and 80% to detect a moderate effect size of 0.56 with 80% power.

Modified intention-to-treat (mITT) analyses were conducted including all participants who received their allocated intervention and who underwent term equivalent brain MRI. This mITT design was necessary given the outcome measures—MRI measures of brain structure and injury—could only be assessed for infants who underwent term-equivalent brain MRI. Per-protocol (PP) analyses were conducted including all participants who received their allocated intervention, underwent term-equivalent brain MRI, and strictly adhered to the protocol based on metabolite status: i.e. comparing brain MRI measures among participants who were randomized to placebo and who were metabolite-negative in maternal blood, urine, and cord blood at delivery (DMEAG and UA = 0 ng/mL), with participants who were randomized to pomegranate juice and who were metabolite-positive (DMEAG and/or UA > 0 ng/mL) in maternal blood, urine, or cord blood at delivery.

Brain injury scores were re-categorized as binary (none vs. ≥ 1) for analysis due to the small number of infants with higher severity scores. Although the clinical importance of mild brain abnormalities still requires further clarification, MRI evidence of mild brain injury has been associated with adverse neurological functioning in the term born infant with mild encephalopathy compared to infants with no brain injury, supporting the premise that MRI-defined injury is a risk factor for subsequent neurodevelopmental challenges^81^. Furthermore, independent measures from the scoring system used in the current study, including brain metrics, have been shown to be related to neurological outcomes in preterm infants, supporting that even a score if 1–2 from such measures may be of neurodevelopmental significance without other overt brain injury^82^. Differences in brain injury by study arm were assessed using risk differences and risk ratios. Exact methods were used to calculate risk measures and their corresponding 95% confidence intervals due to small sample sizes. Group differences in brain volumes and DTI measures were analyzed using a median regression approach due to skewed distributions of the outcomes of interest; models were adjusted for intrafamilial correlation among twins, as well as for potential confounding due to postmenstrual age at MRI, infant sex, maternal BMI at enrollment, and small for gestational age (SGA) status^83^. Differences in compliance and safety measures were evaluated using Wilcoxon rank sum and *χ*^2^ tests (or Fisher’s exact tests for small cell counts, n < 5), as appropriate. Analyses were conducted using SAS version 9.4 (SAS Institute Inc., Cary, N.C.) and STATA 13.1 (StataCorp, Texas, USA).

## Supplementary Information

Supplementary Information 1.

## Data Availability

The datasets generated during and/or analyzed during the current study are not publicly available because we had not previously sought IRB approval for public sharing of participant data as part of our informed consent. However, the de-identified minimal raw dataset is available from the corresponding author upon reasonable request.
