# Supplementary material for: A randomized controlled trial investigating the impact of maternal dietary supplementation with pomegranate juice on brain injury in infants with IUGR
Source: Sci Rep. 2021 Feb 11;11:3569. doi: 10.1038/s41598-021-82144-0 (PMC7878922; doi:10.1038/s41598-021-82144-0)
Supplement: Supplementary file 1 — Supplementary Information 1. [file 41598_2021_82144_MOESM1_ESM.pdf]

A randomized controlled trial investigating the impact of maternal dietary supplementation with pomegranate juice on brain injury in infants with IUGR

Madeline M ROSS[1], Sara CHERKERZIAN, ScD[1], Nicole D MIKULIS[1], Daria TURNER[1], Julian ROBINSON, MD[2], Terrie E INDER, MBChB, MD[1], Lillian G MATTHEWS, PhD[1,3,4*]

[1]Department of Pediatric Newborn Medicine, Brigham and Women's Hospital, Harvard Medical School, Boston, Massachusetts

[2]Department of Obstetrics, Brigham and Women's Hospital, Harvard Medical School, Boston, Massachusetts

[3]Turner Institute for Brain and Mental Health, Monash University, Melbourne, Australia

[4]Monash Biomedical Imaging, Monash University, Melbourne, Australia

**Corresponding Author*

Supplementary Information

S1 Table. Brain volumes by study arm.

S2 Table. DTI measures by study arm.

S3 Table. Continuous brain injury measures by study arm.

S4 Table. Brain injury scores by study arm – modified intent-to-treat (mITT) analysis.

S5 Table. Brain injury scores by study arm – per protocol (PP) analysis.

S6 Table. Brain injury scores by study arm – per protocol (PP) analysis, excluding infants born to mothers who were metabolite positive at enrollment.

S7 Table. Brain injury and study arm, stratified by obesity status for infants with increased extra-axial space in modified intent-to-treat (mITT) analysis, and dilated ventricles in per protocol (PP) analysis

S1 Table. Brain volumes by study arm

	Group Comparisons							
	**MODIFIED INTENTION-TO-TREAT (Placebo, *n*=27; POM, *n*=24)**				**PER-PROTOCOL (Placebo Metabolite -ve, *n*=16; POM Metabolite +ve, *n*=15)**			
	**Estimate (95% CI)**	**SE**	**z**	***P*-value**	**Estimate (95% CI)**	**SE**	**z**	***P*-value**
GM	5.46 (-8.26, 19.19)	7.00	0.78	0.435	4.36 (-17.31, 26.03)	11.06	0.39	0.693
WM	-6.62 (-14.35, 1.10)	3.94	-1.68	0.093	3.49 (-14.30, 21.29)	9.08	0.38	0.700
CSF	-2.14 (-16.22, 11.93)	7.18	-0.30	0.765	-8.02 (-27.34, 11.30)	9.86	-0.81	0.416
DGM	0.19 (-1.61, 1.99)	0.92	0.21	0.837	0.45 (-0.91, 1.81)	0.69	0.65	0.515
Hippocampus	0.09 (-0.83, 1.00)	0.47	0.18	0.854	-0.01 (-0.77, 0.76)	0.39	-0.01	0.988
Amygdala	0.26 (-0.10, 0.63)	0.19	1.41	0.160	-0.08 (-0.44, 0.28)	0.18	-0.45	0.654
Cerebellum	-0.71 (-3.49, 2.07)	1.42	-0.50	0.617	-0.82 (-3.85, 2.21)	1.55	-0.53	0.596
Brainstem	0.00 (-0.78, 0.78)	0.40	0.00	0.999	-0.48 (-2.36, 1.40)	0.96	-0.50	0.615
Total tissue	-9.87 (-34.56, 14.83)	12.60	-0.78	0.434	8.21 (-17.99, 34.41)	13.37	0.61	0.539
ICV	-17.68 (-50.84, 15.48)	16.92	-1.05	0.296	-15.87 (-76.97, 45.22)	31.17	-0.51	0.611
Adjusted for ICV								
GM	5.01 (-5.31, 15.33)	5.26	0.95	0.342	6.42 (-13.24, 26.07)	10.03	0.64	0.522
WM	-3.30 (-9.90, 3.30)	3.37	-0.98	0.327	-2.44 (-14.90, 10.01)	6.36	-0.38	0.701
CSF	-1.17 (-11.01, 8.67)	5.02	-0.23	0.816	-5.04 (-18.05, 7.96)	6.63	-0.76	0.447
DGM	0.18 (-1.42, 1.78)	0.82	0.22	0.823	0.49 (-0.84, 1.82)	0.68	0.72	0.473
Hippocampus	0.05 (-0.81, 0.91)	0.44	0.12	0.903	-0.11 (-0.79, 0.58)	0.35	-0.30	0.762
Amygdala	0.15 (-0.32, 0.62)	0.24	0.61	0.542	-0.09 (-0.41, 0.24)	0.17	-0.52	0.607
Cerebellum	-1.01 (-4.00, 1.97)	1.52	-0.67	0.505	-0.27 (-3.57, 3.04)	1.69	-0.16	0.875
Brainstem	0.00 (-1.10, 1.11)	0.56	0.00	0.998	0.03 (-1.51, 1.58)	0.79	0.04	0.968
Total tissue	1.17 (-8.67, 11.01)	5.02	0.23	0.816	5.04 (-7.96, 18.05)	6.63	0.76	0.447

Group comparisons evaluated using median regression adjusted for intrafamilial correlation among twins, as well as for potential confounding by maternal BMI at enrollment, PMA at MRI scan, infant sex and SGA status (placebo = reference).

GM – cortical grey matter; CSF – cerebrospinal fluid; DGM – deep grey matter; ICV – intracranial volume; POM – pomegranate; WM – white matter

S2 Table. DTI measures by study arm

		Group Comparisons							
		MODIFIED INTENTION-TO-TREAT (Placebo, *n*=19; POM, *n*=18)				PER-PROTOCOL (Placebo Metabolite –ve, *n*=8; POM Metabolite +ve, *n*=11)			
**Tract**		**Estimate (95% CI)**	**SE**	**z**	***P*-value**	**Estimate (95% CI)**	**SE**	**z**	***P*-value**
L ALIC	FA	-0.01 (-0.05, 0.04)	0.02	-0.37	0.711	0.05 (-0.01, 0.10)	0.03	1.76	0.079
	MD	-0.09 (-0.39, 0.21)	0.15	-0.58	0.565	0.66 (-0.62, 1.94)	0.66	1.01	0.314
	AD	0.19 (-1.04, 1.41)	0.63	0.30	0.763	1.17 (-0.93, 3.26)	1.07	1.09	0.276
	RD	0.18 (-0.23, 0.58)	0.21	0.84	0.399	0.38 (-0.58, 1.33)	0.49	0.77	0.442
R ALIC	FA	-0.02 (-0.08, 0.04)	0.03	-0.65	0.515	0.03 (-0.02, 0.08)	0.03	1.27	0.203
	MD	0.13 (-0.25, 0.51)	0.20	0.66	0.507	-0.24 (-0.49, 0.00)	0.12	-1.97	0.049[1]
	AD	0.00 (-1.11, 1.10)	0.56	0.00	0.999	0.17 (-1.42, 1.76)	0.81	0.21	0.833
	RD	0.19 (-0.32, 0.71)	0.26	0.74	0.461	-0.38 (-1.45, 0.69)	0.55	-0.70	0.485
L PLIC	FA	-0.02 (-0.07, 0.03)	0.03	-0.67	0.504	0.04 (-0.03, 0.11)	0.04	1.01	0.315
	MD	0.17 (-0.27, 0.60)	0.22	0.76	0.444	-0.25 (-1.66, 1.15)	0.72	-0.35	0.725
	AD	-0.20 (-1.10, 0.70)	0.46	-0.44	0.661	0.61 (-0.63, 1.85)	0.63	0.96	0.336
	RD	0.15 (-0.45, 0.75)	0.31	0.48	0.632	-0.44 (-1.52, 0.63)	0.55	-0.81	0.419
R PLIC	FA	-0.02 (-0.07, 0.02)	0.02	-1.00	0.319	0.03 (-0.07, 0.14)	0.06	0.62	0.537
	MD	0.17 (-0.19, 0.52)	0.18	0.91	0.364	-0.24 (-1.24, 0.76)	0.51	-0.47	0.636
	AD	-0.20 (-1.09, 0.68)	0.45	-0.45	0.652	0.99 (-2.78, 4.75)	1.92	0.51	0.607
	RD	0.49 (-0.16, 1.14)	0.33	1.48	0.138	-0.34 (-1.51, 0.92)	0.60	-0.57	0.566
L OR	FA	0.01 (-0.03, 0.05)	0.02	0.56	0.573	0.03 (-0.02, 0.07)	0.02	1.16	0.247
	MD	0.11 (-0.78, 1.00)	0.45	0.24	0.812	1.69 (-1.40, 4.78)	1.58	1.07	0.284
	AD	0.54 (-0.65, 1.71)	0.60	0.89	0.374	1.29 (-0.28, 2.86)	0.80	1.61	0.107
	RD	0.02 (-1.03, 1.07)	0.54	0.03	0.977	0.98 (-1.28, 3.24)	1.15	0.85	0.395
R OR	FA	-0.02 (-0.08, 0.03)	0.03	-0.85	0.396	-0.02 (-0.09, 0.05)	0.04	-0.62	0.537
	MD	0.11 (-0.92, 1.14)	0.53	0.21	0.834	0.59 (-1.07, 2.26)	0.85	0.70	0.485
	AD	0.40 (-0.69, 1.50)	0.56	0.72	0.470	0.46 (-0.93, 1.85)	0.71	0.65	0.515
	RD	0.30 (-0.76, 1.36)	0.54	0.56	0.576	0.62 (-1.18, 2.41)	0.92	0.67	0.501
L Frontal	FA	0.00 (-0.04, 0.04)	0.02	0.14	0.891	-0.02 (-0.09, 0.05)	0.03	-0.61	0.543
	MD	-0.76 (-1.97, 0.46)	0.62	-1.22	0.223	0.14 (-1.97, 2.24)	1.08	0.13	0.900
	AD	-0.48 (-1.72, 0.75)	0.63	-0.77	0.442	-0.10 (-2.51, 2.30)	1.23	-0.08	0.932
	RD	-0.98 (-2.60, 0.64)	0.83	-1.19	0.234	0.03 (-2.56, 2.62)	1.32	0.02	0.984

R Frontal	FA	0.02 (0.00, 0.05)	0.01	1.66	0.098	0.02 (-0.01, 0.05)	0.02	1.30	0.194
	MD	-0.24 (-1.48, 0.99)	0.63	-0.39	0.699	-0.43 (-3.33, 2.46)	1.48	-0.29	0.770
	AD	0.10 (-0.97, 1.17)	0.55	0.18	0.856	0.09 (-3.45, 3.63)	1.81	0.05	0.960
	RD	-0.32 (-1.73, 1.10)	0.72	-0.44	0.662	-0.45 (-3.14, 2.25)	1.37	-0.33	0.745
L Occipital	FA	-0.01 (-0.04, 0.01)	0.01	-1.23	0.217	-0.03 (-0.06, 0.00)	0.01	-2.06	0.039[1]
	MD	-0.29 (-1.56, 0.99)	0.65	-0.44	0.661	-1.36 (-4.65, 1.93)	1.68	-0.81	0.419
	AD	-0.48 (-1.68, 0.72)	0.61	-0.79	0.429	-2.22 (-5.63, 1.20)	1.74	-1.27	0.203
	RD	-0.26 (-1.27, 0.76)	0.52	-0.50	0.618	-1.49 (-4.69, 1.71)	1.63	-0.91	0.362
R Occipital	FA	-0.01 (-0.03, 0.02)	0.01	-0.45	0.652	0.01 (0.00, 0.02)	0.01	1.30	0.192
	MD	0.28 (-0.30, 0.87)	0.30	0.95	0.340	0.04 (-2.98, 3.06)	1.54	0.03	0.980
	AD	0.33 (-1.12, 1.77)	0.74	0.44	0.659	0.21 (-2.50, 2.93)	1.39	0.15	0.877
	RD	0.19 (-0.43, 0.80)	0.32	0.59	0.554	-0.03 (-2.82, 2.75)	1.42	-0.02	0.981

Group comparisons evaluated using median regression adjusted for intrafamilial correlation among twins, as well as for potential confounding by maternal BMI at enrollment, PMA at MRI scan, infant sex and SGA status (placebo = reference).

[1] Did not survive FDR correction for multiple comparisons

AD – axial diffusivity; ALIC – anterior limb of internal capsule; FA – fractional anisotropy; Frontal – frontal lobe; L – left; OR – optic radiation; MD – mean diffusivity; Occipital – occipital lobe; PLIC – posterior limb of internal capsule; POM – pomegranate; R – right; RD – radial diffusivity; MD/AD/RD values are $x10^{-4}$

S3 Table. Continuous brain injury measures by study arm

							**Group Comparisons**							
	**Placebo (*n*=31)**			**POM (*n*=25)**			**MODIFIED INTENTION-TO-TREAT[1]**				**MODIFIED INTENTION-TO-TREAT[2]**			
	**25th**	**50th**	**75th**	**25th**	**50th**	**75th**	**Estimate (95% CI)**	**SE**	**z**	***P-value***	**Estimate (95% CI)**	**SE**	**z**	***P-value***
DGM *(cm^2)*	11.25	12.10	13.20	11.50	12.22	12.60	0.12 (-0.82, 1.06)	0.48	0.25	0.803	0.33 (-0.95, 1.61)	0.65	0.51	0.612
BPD *(mm)*	78.07	82.01	88.83	75.80	79.50	83.70	-2.51 (-7.75, 2.73)	2.67	-0.94	0.348	1.63 (-4.32, 7.59)	3.04	0.54	0.591
IHD *(mm)*	1.80	2.56	4.00	0.97	1.96	2.40	-0.60 (-1.52, 0.32)	0.47	-1.28	0.200	-0.51 (-1.44, 0.42)	0.47	-1.08	0.280
rLVD *(mm)*	4.57	5.80	6.90	5.00	5.33	6.32	-0.47 (-1.67, 0.74)	0.61	-0.76	0.447	0.71 (-0.83, 2.25)	0.79	0.91	0.364
lLVD *(mm)*	4.76	5.60	7.90	5.00	5.70	6.94	0.10 (-1.23, 1.43)	0.68	0.15	0.883	0.34 (-1.17, 1.85)	0.77	0.45	0.655
TCD *(mm)*	50.23	53.37	56.40	49.63	51.50	53.40	-1.87 (-4.62, 0.88)	1.40	-1.33	0.182	0.54 (-3.53, 4.61)	2.08	0.26	0.794
	**Placebo Metabolite –ve (*n*=17)**			**POM Metabolite +ve (*n*=16)**			**PER PROTOCOL[1]**				**PER PROTOCOL[2]**			
	**25th**	**50th**	**75th**	**25th**	**50th**	**75th**	**Estimate (95% CI)**	**SE**	**z**	***P-value***	**Estimate (95% CI)**	**SE**	**z**	***P-value***
DGM *(cm^2)*	10.90	12.10	13.30	11.88	12.40	12.85	0.30 (-1.04, 1.64)	0.68	0.44	0.661	1.22 (-0.45, 2.88)	0.85	1.43	0.152
BPD *(mm)*	79.71	83.71	88.83	74.96	82.69	83.68	-1.21 (-8.45, 6.03)	3.69	-0.33	0.743	1.12 (-6.47, 8.71)	3.87	0.29	0.772
IHD *(mm)*	2.30	2.63	4.22	0.82	1.55	2.54	-0.93 (-2.22, 0.35)	0.66	-1.42	0.154	-0.68 (-1.81, 0.45)	0.58	-1.18	0.240
rLVD *(mm)*	4.49	5.22	6.90	4.60	5.20	5.89	-0.02 (-1.95, 1.91)	0.99	-0.02	0.985	1.13 (-0.87, 3.12)	1.01	1.10	0.269
lLVD *(mm)*	4.76	5.55	8.52	4.92	5.68	6.35	0.12 (-1.51, 1.74)	0.83	0.14	0.890	-0.90 (-2.90, 1.10)	1.02	-0.88	0.380
TCD *(mm)*	49.63	53.90	56.31	49.00	52.09	54.17	-1.22 (-5.80, 3.36)	2.34	-0.52	0.602	1.73 (-4.31, 7.76)	3.08	0.56	0.575

[1] Group comparisons evaluated using median regression adjusted for intrafamilial correlation among twins (placebo = reference).

[2] Group comparisons evaluated using median regression adjusted for intrafamilial correlation among twins, as well as for potential confounding by maternal BMI at enrollment, PMA at MRI scan, infant sex and SGA status (placebo = reference).

BPD – biparietal diameter; DGM – deep grey matter (total basal ganglia area); IHD – interhemispheric distance; lLVD – left lateral ventricle diameter; rLVD – right lateral ventricle diameter; POM – pomegranate; TCD – transverse cerebellar diameter

S4 Table. Brain injury scores by study arm – modified intent-to-treat (mITT) analysis.

		Placebo (*n*=31)		POM (*n*=26)	
	**Score**	***n***	***%***	***n***	***%***
**WM cystic lesions**					
	**0**	31	100.0	26	100.0
	**≥ 1**	0	0.0	0	0.0
**WM focal signal abnormality**					
	**0**	23	74.2	19	73.1
	**1**	5	16.1	4	15.4
	**2**	3	9.7	3	11.5
**WM myelination delay**					
	**0**	22	71.0	22	84.6
	**1**	9	29.0	4	15.4
**WM volume reduction[1]**					
	**0**	24	77.4	18	72.0
	**1**	6	19.4	3	12.0
	**2**	1	3.2	3	12.0
	**3**	0	0.0	1	4.0
**Dilated ventricles[1]**					
	**0**	21	67.7	20	80.0
	**1**	5	16.1	3	12.0
	**2**	5	16.1	2	8.0
**IVH**					
	**0**	30	96.8	26	100.0
	**1**	1	3.2	0	0.0
**GM signal abnormality**					
	**0**	31	100.0	26	100.0
	**> 0**	0	0.0	0	0.0
**GM gyral maturation**					
	**0**	24	77.4	24	92.3
	**1**	6	19.4	2	7.7
	**2**	1	3.2	0	0.0
**DGM signal abnormality**					
	**0**	30	96.8	26	100.00
	**1**	1	3.2	0	0.00
**DGM volume reduction[1]**					
	**0**	31	100.0	25	100.0
	**1**	0	0.0	0	0.0
**Increased extra-axial space[1]**					
	**0**	23	74.2	25	100.0
	**1**	4	12.9	0	0.0
	**2**	4	12.9	0	0.0
**CB signal abnormality**					

	**0**	29	93.6	26	100.0
	**1**	2	6.5	0	0.0
**CB volume reduction[1]**					
	**0**	24	77.4	16	64.0
	**1**	6	19.4	8	32.0
	**2**	1	3.2	1	4.0
**Total WM score**					
	**0**	9	29.0	13	50.0
	**1**	7	22.6	2	7.7
	**2**	9	29.0	5	19.2
	**3**	6	19.4	4	15.4
	**4**	0	0.0	1	3.9
	**5**	0	0.0	1	3.9
**Total DGM Score**					
	**0**	30	96.8	26	100.0
	**1**	1	3.2	0	0.0
**Total GM Score**					
	**0**	20	64.5	24	92.3
	**1**	5	16.1	2	7.7
	**2**	3	9.7	0	0.0
	**3**	3	9.7	0	0.0
**Total CB Score**					
	**0**	23	74.2	17	65.4
	**1**	7	22.6	8	30.8
	**2**	0	0.0	1	3.9
	**3**	1	3.2	0	0.0
**Global Abnormality Score**					
	**0**	5	16.1	11	42.3
	**1**	6	19.4	3	11.5
	**2**	8	25.8	3	11.5
	**3**	5	16.1	5	19.2
	**4**	2	6.5	0	0.0
	**5**	2	6.5	3	115
	**6**	2	6.5	1	3.9
	**8**	1	3.2	0	0.0

[1] Metric-based data missing for 1 POM infant, percentages calculated for total number of infants for whom data were available

CB – cerebellum; DGM – deep grey matter; GM – cortical grey matter; IVH – intraventricular hemorrhage; POM – pomegranate; WM – white matter

S5 Table. Brain injury scores by study arm – per protocol (PP) analysis.

		Placebo Metabolite –ve (*n*=17)		POM Metabolite +ve (*n*=17)	
	**Score**	***n***	**%**	***n***	**%**
**WM cystic lesions**					
	**0**	17	100.0	17	100.0
	**≥ 1**	0	0.0	0	0.0
**WM focal signal abnormality**					
	**0**	12	70.6	15	88.2
	**1**	3	17.7	1	5.9
	**2**	2	11.8	1	5.9
**WM myelination delay**					
	**0**	12	70.6	16	94.1
	**1**	5	29.4	1	5.9
**WM volume reduction[1]**					
	**0**	14	82.4	12	75.0
	**1**	3	17.7	1	6.3
	**2**	0	0.0	3	18.8
	**3**	0	0.0	0	0.00
**Dilated ventricles[1]**					
	**0**	10	58.8	15	93.8
	**1**	5	29.4	1	6.3
	**2**	2	11.8	0	0.0
**IVH**					
	**0**	16	94.1	17	100.0
	**1**	1	5.9	0	0.0
**GM signal abnormality**					
	**0**	17	100.0	17	100.0
	**≥ 1**	0	0.0	0	0.0
**GM gyral maturation**					
	**0**	12	70.6	17	100.0
	**1**	4	23.5	0	0.0
	**2**	1	5.9	0	0.0
**DGM signal abnormality**					
	**0**	16	94.1	17	100.0
	**1**	1	5.9	0	0.0
**DGM volume reduction[1]**					
	**0**	17	100.0	16	100.0
	**1**	0	0.0	0	0.0
**Increased extra-axial space[1]**					
	**0**	12	70.6	16	100.0
	**1**	1	5.9	0	0.0
	**2**	4	23.5	0	0.0
**CB signal abnormality**					
	**0**	16	94.1	17	100.0

	**1**	1	5.9	0	0.0
**CB volume reduction**[1]					
	**0**	11	64.7	10	62.5
	**1**	5	29.4	5	31.3
	**2**	1	5.9	1	6.3
**Total WM Score**					
	**0**	3	17.7	11	64.7
	**1**	6	35.3	2	11.8
	**2**	6	35.3	2	11.8
	**3**	2	11.8	2	11.8
	**4**	0	0.0	0	0.0
	**5**	0	0.0	0	0.0
**Total DGM Score**					
	**0**	16	94.1	17	100.0
	**1**	1	5.9	0	0.0
**Total GM Score**					
	**0**	10	58.8	17	100.0
	**1**	2	11.8	0	0.0
	**2**	2	11.8	0	0.0
	**3**	3	17.7	0	0.0
**Total CB Score**					
	**0**	11	64.7	11	64.7
	**1**	5	29.4	5	29.4
	**2**	0	0.0	1	5.9
	**3**	1	5.9	0	0.0
**Global Abnormality Score**					
	**0**	0	0.0	9	52.9
	**1**	6	35.3	3	17.7
	**2**	4	23.5	1	5.9
	**3**	2	11.8	3	17.7
	**4**	1	5.9	0	0.0
	**5**	1	5.9	1	5.9
	**6**	2	11.8	0	0.0
	**8**	1	5.9	0	0.0

[1] Metric-based data missing for 1 POM infant, percentages calculated for total number of infants for whom data were available

CB – cerebellum; DGM – deep grey matter; GM – cortical grey matter; IVH – intraventricular hemorrhage; POM – pomegranate; WM – white matter

S6 Table. Brain injury scores by study arm – per protocol (PP) analysis, excluding infants born to mothers who were metabolite positive at enrollment

		Placebo Metabolite –ve *n*=16		POM Metabolite +ve *n*=9	
	**Score**	***n***	**%**	***n***	**%**
**WM cystic lesions**					
	**0**	16	100.0	9	100.0
	**≥ 1**	0	0.0	0	0.0
**WM focal signal abnormality**					
	**0**	12	75.0	9	100.0
	**1**	2	12.5	0	0.0
	**2**	2	12.5	0	0.0
**WM myelination delay**					
	**0**	12	75.0	8	88.9
	**1**	4	25.0	1	11.1
**WM volume reduction[1]**					
	**0**	13	81.3	6	75.0
	**1**	3	18.8	1	12.5
	**2**	0	0.0	1	12.5
	**3**	0	0.0	0	0.0
**Dilated ventricles[1]**					
	**0**	9	56.3	8	100.0
	**1**	5	31.3	0	0.0
	**2**	2	12.5	0	0.0
**IVH**					
	**0**	15	93.8	9	100.0
	**1**	1	6.3	0	0.0
**GM signal abnormality**					
	**0**	16	100.0	9	100.0
	**≥ 1**	0	0.0	0	0.0
**GM gyral maturation**					
	**0**	11	68.8	9	100.0
	**1**	4	25.0	0	0.0
	**2**	1	6.3	0	0.0
**Increased extra-axial space[1]**					
	**0**	11	68.8	8	100.0
	**1**	1	6.3	0	0.0
	**2**	4	25.0	0	0.0
**DGM signal abnormality**					
	**0**	15	93.8	9	100.0
	**1**	1	6.3	0	0.0
**DGM volume reduction[1]**					
	**0**	16	100.0	8	100.0
	**1**	0	0.0	0	0.0

**CB signal abnormality**					
	**0**	15	93.8	9	100.0
	**1**	1	6.3	0	0.0
**CB volume reduction[1]**					
	**0**	10	62.5	5	62.5
	**1**	5	31.3	2	25
	**2**	1	6.3	1	12.5
**Total WM Score**					
	**0**	3	18.8	7	77.8
	**1**	6	37.5	1	11.1
	**2**	5	31.3	0	0.0
	**3**	2	12.5	1	11.1
	**4**	0	0.0	0	0.0
	**5**	0	0.0	0	0.0
**Total DGM Score**					
	**0**	15	93.8	9	100.0
	**1**	1	6.3	0	0.0
**Total GM Score**					
	**0**	9	56.3	9	100.0
	**1**	2	12.5	0	0.0
	**2**	2	12.5	0	0.0
	**3**	3	18.8	0	0.0
**Total CB Score**					
	**0**	10	62.5	6	66.7
	**1**	5	31.3	2	22.2
	**2**	0	0.0	1	11.1
	**3**	1	6.3	0	0.0
**Global Abnormality Score**					
	**0**	0	0.0	6	66.7
	**1**	6	37.5	1	11.1
	**2**	3	18.8	1	11.1
	**3**	2	12.5	0	0.0
	**4**	1	6.3	0	0.0
	**5**	1	6.3	1	11.1
	**6**	2	12.5	0	0.0
	**8**	1	6.3	0	0.0

[1] Metric-based data missing for 1 POM infant, percentages calculated for total number of infants for whom data were available

CB – cerebellum; DGM – deep grey matter; GM – cortical grey matter; IVH – intraventricular hemorrhage; POM – pomegranate; WM – white matter

S7 Table. Brain injury and study arm, stratified by obesity status for infants with increased extra-axial space in modified intent-to-treat (mITT) analysis, and dilated ventricles in per protocol (PP) analysis

**mITT – Juice association with extra-axial space, by obesity status[1]**				
	**POM (*n*=25)[2]**		**Placebo (*n*=31)**	
	**Obese (*n*=6)**	**Non-Obese (*n*=19)**	**Obese (*n*=16)**	**Non-Obese (*n*=15)**
Increased extra-axial space, *n (%)*	0 (0)	0 (0)	6 (37.5)	2 (13.3)
**PP – Juice association with enlarged ventricles, by obesity status[1]**				
	**POM Metabolite +ve (*n*=16)[2]**		**Placebo Metabolite –ve (*n*=17)**	
	**Obese (*n*=4)**	**Non-obese (*n*=12)**	**Obese (*n*=10)**	**Non-obese (*n*=7)**
Dilated ventricles, *n (%)*	1 (25)	0 (0)	6 (60)	1 (14.3)

[1] Percentages shown are for infants with injury only, stratified by obesity status (cutoff, BMI > 30)

[2] Metric-based data missing for 1 POM infant, percentages calculated for total number of infants for whom data were available
